# Differentiating Gliosarcoma from Glioblastoma: A Novel Approach Using PEACE and XGBoost to Deal with Datasets with Ultra-High Dimensional Confounders

**DOI:** 10.3390/life14070882

**Published:** 2024-07-16

**Authors:** Amir Saki, Usef Faghihi, Ismaila Baldé

**Affiliations:** 1 Département de Mathématiques et d’Informatique, Université du Québec à Trois-Rivières, Trois-Rivières, QC G8Z 4M3, Canada; amir.saki@uqtr.ca; 2 Département de Mathématiques et de Statistique, Faculté des Sciences, Université de Moncton, Moncton, NB E1A3E9, Canada; ismaila.balde@umoncton.ca

**Keywords:** Probabilistic Easy Variational Causal Effect (PEACE), causal inference, XGBoost, gliosarcoma, glioblastoma

## Abstract

In this study, we used a recently developed causal methodology, called Probabilistic Easy Variational Causal Effect (PEACE), to distinguish gliosarcoma (GSM) from glioblastoma (GBM). Our approach uses a causal metric which combines Probabilistic Easy Variational Causal Effect (PEACE) with the XGBoost, or eXtreme Gradient Boosting, algorithm. Unlike prior research, which often relied on statistical models to reduce dataset dimensions before causal analysis, our approach uses the complete dataset with PEACE and the XGBoost algorithm. PEACE provides a comprehensive measurement of direct causal effects, applicable to both continuous and discrete variables. Our method provides both positive and negative versions of PEACE together with their averages to calculate the positive and negative causal effects of the radiomic features on the variable representing the type of tumor (GSM or GBM). In our model, PEACE and its variations are equipped with a degree d which varies from 0 to 1 and it reflects the importance of the rarity and frequency of the events. By using PEACE with XGBoost, we achieved a detailed and nuanced understanding of the causal relationships within the dataset features, facilitating accurate differentiation between GSM and GBM. To assess the XGBoost model, we used cross-validation and obtained a mean accuracy of 83% and an average model MSE of 0.130. This performance is notable given the high number of columns and low number of rows (code on GitHub).

## 1. Introduction

Glioblastoma (GBM) stands as a major form of brain cancer, originating in glial cells [1,2]. Among its variants, gliosarcoma (GSM) is classified by the World Health Organization (WHO) as a subtype of GBM, necessitating accurate differentiation between GSM and GBM to implement effective treatment strategies [1,3]. Radiomics, a field that uses imaging data for analysis, has shown promise in distinguishing different types of central nervous system tumors [4,5,6]. However, a significant challenge in distinguishing between GSM and GBM is dealing with high-dimensional datasets that have a limited number of samples. To tackle this problem, recent advancements in the field have led to the development of machine learning algorithms capable of handling the high-dimensional datasets.

For instance, Qian et al. [7] sought to identify an optimal machine learning algorithm for differentiating GSM from GBM using a radiomics-based approach. Their dataset comprised 1303 radiomics features from 183 patients, highlighting the issue of data dimensionality surpassing the sample size, known as the “curse of dimensionality”. This phenomenon necessitates sophisticated variable selection algorithms to improve model accuracy and interpretability.

So far, many researchers have attempted to tackle the problem of the curse of dimensionality. Jaman et al. [8] addressed the issues related to dimension reduction and collinearity using penalized regression techniques such as SCAD (smoothly clipped absolute deviation) for variable selection. This method mitigates collinearity issues by selecting a subset of relevant variables and shrinking the coefficients of less relevant ones to zero, thereby reducing the effective dimensionality of the model and addressing potential collinearity problems. Qian Gao et al. [9] focused on evaluating propensity score (PS) methods for causal inference in high-dimensional datasets, particularly in observational studies with many covariates. To do so, they used methods such as LASSO (Least Absolute Shrinkage and Selection Operator) for dimension reduction before using propensity score (PS) methods to balance the covariates between treatment groups. Ziyan Zhang et al. [10] used penalized regression methods, specifically the Least Absolute Shrinkage and Selection Operator (LASSO) and elastic net regularization, to address the high-dimensional dataset problem. These techniques are used for variable selection and regularization to reduce the number of features and avoid issues related to collinearity and overfitting.

However, gaps remain in addressing the full dataset without dimension reduction methods, and the challenge of ultra-high dimensional data, where the number of features is much larger than the sample size, remains a significant hurdle, leading to potential collinearity issues [7,8,9,11,12,13].

To the best of our knowledge, our study is the first to use a novel causal approach to analyze radiomics datasets without employing any dimension reduction algorithms or techniques. Our causal approach is equipped with a causal metric called Probabilistic Easy Variational Causal Effect (PEACE) and its average version which is called MEAN PEACE [14,15]. PEACE, derived from the principles of total variation [16], offers a robust framework for measuring the direct causal effect of one variable on another, for both continuous and discrete data types. A unique property of PEACE is that it has two variations capable of calculating both positive and negative causal effects of exposures by considering the likelihood of transitioning from one event to the next. It is effective in handling causal inference from both detailed (micro) and broad (macro) levels of causal analysis. PEACE can adapt to a wide range of probability density values and integrates the occurrence frequency and rarity of the event, providing a deeper understanding of causal relationships between variables.

Causal models excel in reasoning but are unable to learn, whereas machine learning causal models excel in reasoning but are unable to learn; machine learning algorithms also excel in learning but have limited reasoning capabilities [17]. To use the strengths of both, we used PEACE, with XGBoost [18], or eXtreme Gradient Boosting, which is a high-performance implementation of gradient boosting known for its speed and effectiveness in machine learning challenges and applications to identify GSM from GBM. 

## 2. Causal Reasoning

In causal reasoning, we seek to understand how or why something happened and the relationship between causes and their effects. We examine these relationships to comprehend the underlying mechanisms and reasons behind events. To calculate the causes of an event, current causal models often use Individual or Average Treatment Effect (I-ATE). For instance, Pearl [19] computed the Average Causal Effect by subtracting the means of the outcomes of the treatment and control groups. Pearl used Directed Acyclic Graphs (DAGs) to visualize and compute the associations or causal relationships between a set of random variables. He also used *do* operators to formalize interventions on the nodes of DAGs in connection with probability theory, the (local) Markov assumption, and other concepts/methods/tools [19].

However, in [14], the authors demonstrated that ATE cannot fully describe causality in the presence of a non-linear relationship between variables. Also, Pearl’s approach to causation does not allow for reasoning in cases with degrees of uncertainty [20]. Since *do* operators cut the relation between two nodes, Pearl’s approach cannot answer gradient questions such as, given that you smoke a little, what is the probability that you have cancer to a certain degree? To solve Pearl’s do operator problem, the authors of [20] used fuzzy logic rules which implement human language nuances such as “*small*” and “*high*” instead of mere zero or one. Furthermore, Janzing [21] showed that at a macro level, Pearl’s causal model works well with situations that are rare, such as rare medical conditions but, at a micro level, fails with bidirectional nodes. The authors of [14] showed that Janzing’s model [22] works well with bidirectional nodes, but fails with situations that are rare [14].

To our knowledge, the existing causal models overlook the rarity and frequency of dataset elements, which are vital for assessing their impact on events. That is why we introduce PEACE which considers the availability, rarity, and frequency of the events as well as the use of interventions for causal inference in the next subsection.

### 2.1. Probabilistic Easy Variational Causal Effect (PEACE)

Total variation [16] quantifies the cumulative changes in a sequence or a function by summing the absolute differences between successive data points, for example, $\left|x_{1}-x_{0}\right|$ in a sequence ${\left(x_{n}\right)}_{n=0}^{\infty }$. In the realm of causal inference, the Probabilistic Easy Variational Causal Effect (PEACE) model [15] utilizes this concept to dissect the positive, negative, and overall effects of features within a dataset on the outcomes, accommodating the dual nature of features that can exhibit both beneficial and damaging effects depending on their prevalence.

The innovative aspect of the PEACE model lies in its incorporation of probabilistic elements, degree of availability, and interventions into the total variation formula, which measures the fluctuations, rarity, and frequency of events to identify their causes. Furthermore, the PEACE formula can be adapted to handle both discrete and continuous variables, using total variation as its foundation [15].

Central to the PEACE model is the parameter *d*, which calibrates the sensitivity of the analysis to changes of both positive and negative PEACE values (see below). The *d* values range between zero and one, where the causal effects of all events are considered with the same importance as low *d* values, and the causal effects of more common occurrences are more pronounced with high *d* values closer to one. This adaptability allows for a comprehensive exploration of potential outcomes across the spectrum of event frequencies.

Assume that $Y=g\left(T,C\right)$, where C represents the set of all covariates including the confounders. The formal representation of the PEACE of degree d for the causal effect of a finite variable T on an outcome Y, considering all possible values $t_{0}<t_{1},\ldots <t_{l}$ of T, is given by

(1) $$\;PEACE_{d}\left(Y;T\right)≔\sum_{i=1}^{l}\left|E\left[g_{in}\left(t_{i}\right)\right]-E\left[g_{in}\left(t_{i-1}\right)\right]\right|\cdot \;P{\left(t_{i}\right)}^{d}\cdot P{\left(t_{i-1}\right)}^{d}$$

where for each T=t, $E\left[g_{in}\left(t\right)\right]$ is the average potential outcome under the intervention T=t, and $P\left(t\right)$ denotes the probability of T taking the value t.

Note that the confounders are factors that could potentially influence both the treatment variable T and the outcome variable Y, and hence, potentially confounding or obscuring the true causal relationship between T and Y.

By considering C, the PEACE model aims to isolate the direct causal effect of T on Y, controlling for the influence of these confounders. This approach enhances the model’s to provide a more accurate and nuanced understanding of the causal relationship between the variables of interest. The presence of C in the formula acknowledges the complexity of real-world data, where multiple interconnected factors may impact the outcome.

In addition to the PEACE formula, which calculates the *total* causal impact of the treatment/exposure variable on the outcome, we can also calculate the *average* causal impacts using the mean PEACE formula, which divides the PEACE value by the sum of the weights (availability values) used in the PEACE formula. Thus,

(2) $$Mean\;PEACE_{d}\left(Y;T\right)≔\;\frac{PEACE_{d}\left(Y;T\right)}{\sum_{i=1}^{l}P{\left(t_{i}\right)}^{d}\cdot P{\left(t_{i-1}\right)}^{d}}$$

### 2.2. Positive and Negative PEACE Explanation

The positive and negative Probabilistic Easy Variational Causal Effect (PEACE) metrics are essential causal metrics in our methodology that quantify the causal impact of a variable on an outcome, taking into account the varying degrees of event occurrences. These metrics are derived from the total variation formula, which measures the cumulative changes within a sequence by summing the absolute differences between successive data points.

#### 2.2.1. Positive PEACE

Positive PEACE focuses on the total positive causal changes in an outcome Y when increasing the value of a treatment or variable T. It is defined mathematically for a sequence of values $t_{0}<t_{1},\ldots <t_{l}$ as follows:

(3) $$PEACE_{d}{\left(Y;T\right)}^{+}≔\sum_{i=1}^{l}{\left(E\left[g_{in}\left(t_{i}\right)\right]\;-\;E\left[g_{in}\left(t_{i-1}\right)\right]\right)}^{+}\cdot P{\left(t_{i}\right)}^{d\;}\cdot P{\left(t_{i-1}\right)}^{d}$$

where

- For each T=t, $E\left[g_{in}\left(t\right)\right]$ represents the average potential outcome after the intervention sets the treatment T to the specific value t.
- The superscript + indicates that only positive differences (indicating an increase in the outcome due to the treatment) are considered.
- P(t) denotes the probability of the treatment taking a specific value t, and d is a parameter that adjusts the weighting based on the degree of event availability.

The mean positive PEACE is naturally defined as well. 

#### 2.2.2. Negative PEACE

Conversely, negative PEACE measures the total negative causal changes when the treatment’s value increases. It is mathematically expressed as

(4) $$PEACE_{d}{\left(Y;T\right)}^{-}≔\sum_{i=1}^{l}{\left(E\left[g_{in}\left(t_{i}\right)\right]-E\left[g_{in}\left(t_{i-1}\right)\right]\right)}^{-}\cdot P{\left(t_{i}\right)}^{d}\;\cdot \;P{\left(t_{i-1}\right)}^{d}$$

In this formula,

- The superscript − signifies that only the negative parts of the differences are considered, focusing on decreases in the outcome as the treatment value increases.
- The rest of the terms mirror those in the positive PEACE formula, with P(t) and d again representing the probabilities and the degree of event availability, respectively.

The MEAN negative PEACE is naturally defined as well.

The positive and negative PEACE metrics provide nuanced insights into the causal dynamics at play, distinguishing between the beneficial and damaging effects of altering a treatment or variable. By considering the degrees of event availability, these metrics allow for a more granular and probabilistically informed understanding of causality, making them particularly useful in complex datasets where a single variable can have multifaceted impacts on an outcome.

## 3. Methodology

We used eXtreme Gradient Boosting (XGBoost) [18] for learning purposes, analysis libraries such as SHapley Additive exPlanations (SHAP) [23], and causal inference functions such as marginal effect and causal inference libraries such as Double ML and our Probabilistic Easy Variational Causal Effect (PEACE).

Before explaining our methodology, we will briefly explain the above machine learning/causal models.

**XGBoost** [18] is built on the gradient boosting framework, where new models are added sequentially to correct the errors made by previous models. It uses decision trees as base learners. XGBoost optimizes the loss function (like log loss for classification problems) by computing the gradient (the direction and magnitude of error) and updating the model accordingly to minimize this loss. It includes L1 (LASSO Regression) and L2 (Ridge Regression) regularization terms in the cost function to control over-fitting, making it robust. XGBoost can automatically learn the best direction to handle missing values during training. Unlike traditional gradient boosting that grows trees greedily, XGBoost grows trees to a max depth and then prunes back branches that have little value, optimizing both computational efficiency and model performance. XGBoost utilizes multi-threading and can be run on distributed computing frameworks like Hadoop, making it extremely fast.

The **Shapley** value [23] is mainly used in game theory. It is used to fairly distribute the total gains (or losses) among players based on their individual contributions to the game. In the context of machine learning, Shapley values are used to interpret predictive models by measuring the contribution of each feature to the prediction of a particular instance. Shapley values do not determine causality. They indicate the importance and contribution of features within the context of a specific model, which may or may not reflect true causal relationships.

**Marginal Effect** offers a way to estimate how changes in individual features impact an outcome. It calculates the marginal causal effects of specific features on an outcome by simulating interventions in a dataset. It uses percentiles to sample unique feature values and computes the resulting mean outcome, optionally applying a logit transformation for logistic regression models. The function then centers the results to capture deviations from the average effect, producing a series of pairs that show how variations in each feature influence the outcome. This approach efficiently quantifies the marginal effects across different feature values.

The **DoubleML** library [24] is a Python package designed for causal inference. It implements two-stage regression techniques to control for confounding variables and derive reliable causal estimates from observational data. Contrary to marginal effects and our PEACE, DoubleML does not calculate feature-specific causal effects; instead, it focuses on estimating the Average Treatment Effect (ATE), which represents the overall effect of the treatment.

### Implementation

**Figure 1:**
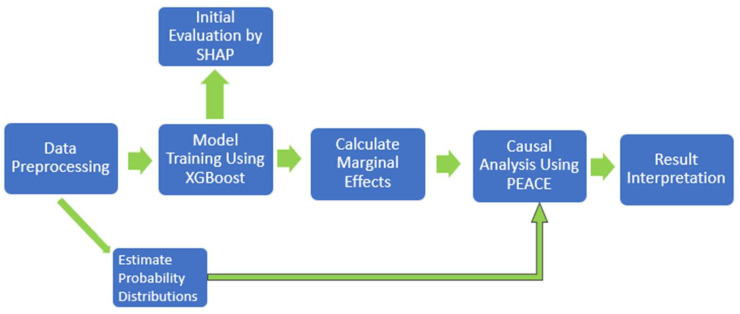
Workflow for PEACE calculation with XGBoost.

Roughly speaking, as shown in Figure 1, we first used the XGBoost algorithm to train a model using our dataset. After the model was trained, we used the SHAP library to interpret the model’s predictions. To do so, we used the code from [25] with some changes such as implementing interventions from marginal effects with certain modifications. The marginal effects function first calculates percentile values for each feature in the dataset. Then, the function applies interventions which allows us to fix certain features at specific values to observe the changes in output explicitly. For each fixed percentile, the code computes the SHAP values using the SHAP Explainer applied to the XGBoost model we trained earlier. These SHAP values are calculated to assess how each feature contributes to the predicted outcome at various fixed levels, which does not reflect the causal impact of each feature. 

To perform causal inference, we then used our Probabilistic Easy Variational Causal Effect (PEACE) to evaluate the changes in the probability distribution of the predicted outcome as a function of varying a feature across its range. The code, which can be found on GitHub, computes PEACE values for each feature by calculating the positive and negative effects of increasing or decreasing a feature’s value, respectively. It then plots these PEACE values against a parameter *d*, which represents different thresholds or levels of change which represents how changes to a specific variable can lead to changes in the outcome, both positively and negatively. 

## 4. Dataset Description

We used the radiomics data studied in Qian et al. [7]. The dataset contained a sample size of *n* = 183 patients including 100 with GBM and 83 with GSM with 1303 radiomic features extracted from MRI images. In our study, we considered each radiomic feature and “Edema” (Yes: 1/No: 0) as the exposure variable (treatment) and the others as confounders/mediators. We took Y (Y = 1 if the patient had gliosarcoma and Y = 0 if the patient had glioblastoma) as the outcome variable. We then calculated the PEACE, the mean PEACE, and their positive and negative variations to determine the causal impacts of each variable on Y.

## 5. Results

In this section, we first show the result from applying SHAP to the dataset. We then use the marginal effect function to calculate the average causal effect of the intervention/treatment. The results from the marginal effect function are compared to SHAP values. Then, we apply the DoubleML library to the dataset and compare its results with SHAP. Finally, we present the PEACE and mean PEACE results and compare the positive and negative PEACE outputs in terms of feature selection and accuracy. The objective was to identify the key radiomics features with the highest direct causal impact on distinguishing between GSM and GBM.

### 5.1. SHAP

**Figure 2:**
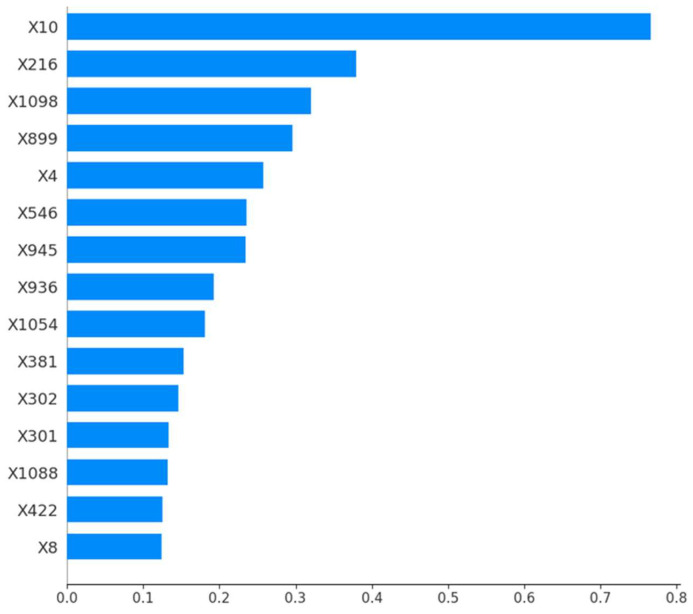
Average impact (the mean absolute SHAP values) of top features on the model’s predictions. Features with higher values have a larger impact.

In Figure 2, the SHAP values represent the average impact of each feature on the model’s output magnitude. The feature X10 which corresponds to the column named ‘original_shape_Sphericity’ in the dataset, showed the highest impact, with a mean SHAP value close to 0.8, indicating a large influence on the model output. This is followed by features X216, X1098, and X899, each displaying values slightly below 0.5. The rest of the features have SHAP values below 0.3, suggesting comparatively lower influences on the model output. 

### 5.2. Marginal Effect

**Figure 3:**
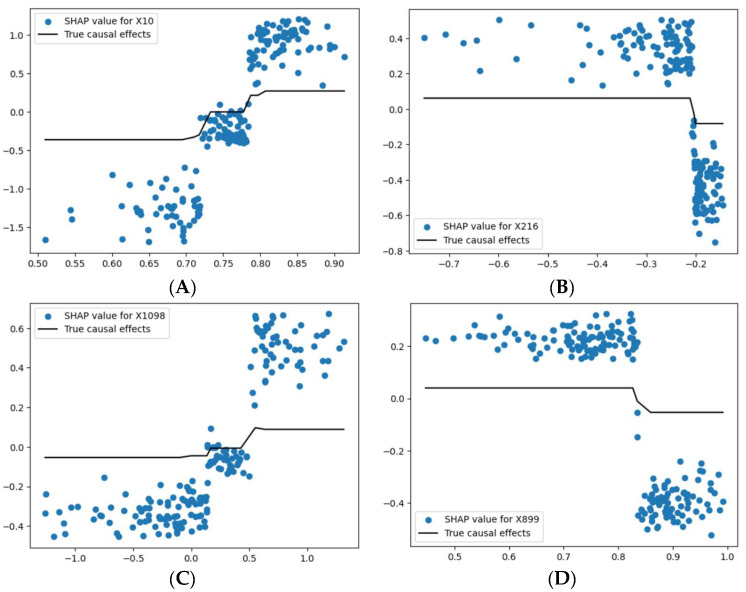
(**A**) Represents SHAP values versus true causal effect values for feature X10, (**B**) Represents SHAP values versus true causal effect values for feature X216, (**C**) Represents SHAP values versus true causal effect values for feature X1098, (**D**) Represents SHAP values versus true causal effect values for feature X899.

Figure 3 demonstrates the four top values obtained using the marginal effect function with their corresponding SHAP values. 

In Figure 3A, the SHAP values for feature X10 exhibited a sharp increase between the values of 0.80 and 0.85, as shown in the scatter plot. The SHAP values were relatively lower and more varied when X10 was below this threshold. The black line represents the true causal effects, which showed a sudden step up at around 0.85, indicating a threshold effect where X10 significantly influenced the model’s output after this point.

For the other variables, X216, X1098, and X899, as seen in Figure 3B–D, respectively, the true marginal effects values were smaller in comparison to X10. Specifically, in Figure 3B–D, the marginal effect values were mostly around zero, suggesting that while changes in these features did affect the model’s predictions, their overall impact was less pronounced than that of X10.

### 5.3. DoubleML

In the second part of our experiment, we used the DoubleML library [24], a Python package designed for analyzing causal inference. 

**Figure 4:**
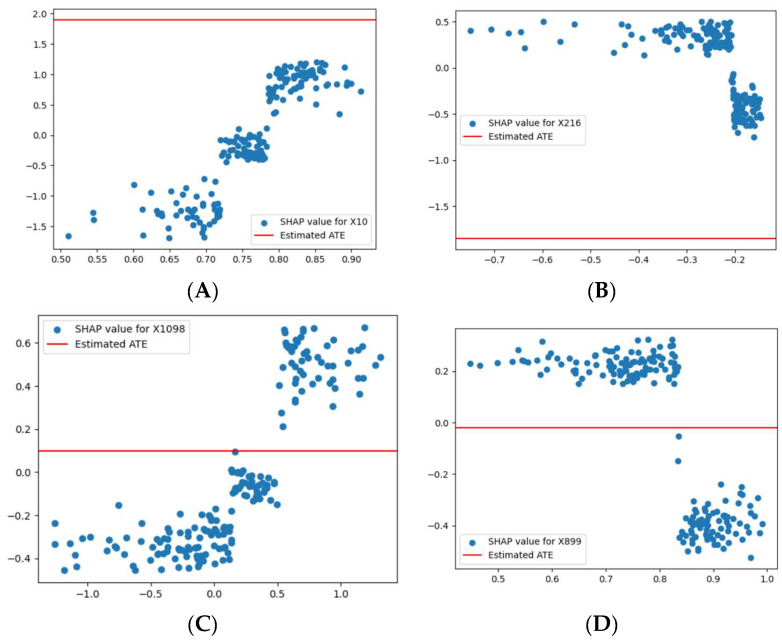
(**A**) Represents SHAP values versus estimated Average Treatment Effect (ATE) value for feature X10, (**B**) Represents SHAP values versus estimated ATE value for feature X216, (**C**) Represents SHAP values versus estimated ATE value for feature X1098, (**D**) Represents SHAP values versus estimated ATE value for feature X899.

In Figure 4, the SHAP values for each feature (X10, X216, X1098, and X899) were the same as those in Figure 3. However, in Figure 4, the Average Treatment Effect (ATE) is represented as a constant horizontal red line for all variables.

Figure 4A (X10): The constant ATE line was slightly lower than the highest SHAP values, indicating a modest average impact of X10 on the outcome.

Figure 4B (X216): Despite the variability in SHAP values for X216, the constant ATE line suggests that, on average, X216’s impact on the model’s outcome was minimal.

Figure 4C (X1098): The constant ATE value for X1098 was slightly lower than the highest SHAP values, indicating a limited overall average causal effect.

Figure 4D (X899): The constant ATE value for X899 remained slightly below the highest SHAP values, suggesting a minimal average influence on the model’s outcome.

Overall, the constant ATE lines in Figure 4 indicate that the estimated general average marginal impact of these features (X10, X216, X1098, and X899) on the model’s outcome was limited and did not vary significantly with changes in their values. This suggests that while these features might affect predictions in specific instances or contexts, the constant causal impact fails to capture potential causal changes across the entire domain of variables.

### 5.4. Mean Positive PEACE

**Figure 5:**
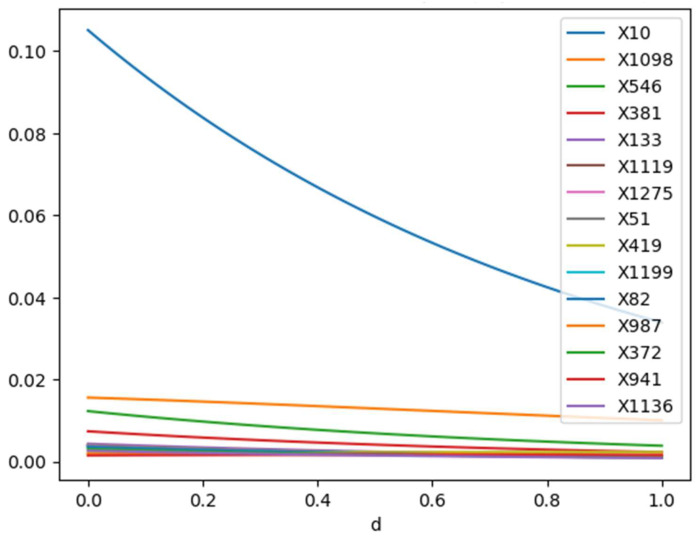
Mean positive PEACE values for the top 15 features for degrees d between 0 and 1.

Figure 5 shows the top 15 mean PEACE values where X10 (the blue line) corresponds to ‘original_shape_Sphericity’ in the dataset; it had the highest values compared to other variables. The other fourteen values were very small compared to X10. In Figure 5, for all features, as *d* increased from 0 to 1, the mean positive PEACE values decreased. This indicates that the causal effects corresponding to more probable regions were not significantly greater than those in other regions. 

This could suggest that higher density regions might be associated with more stable, less variable influences on the outcome, while lower density regions (lower ‘*d’* values), which emphasize rare or extreme cases, might exhibit stronger causal effects.

With regard to magnitude, the scales of the PEACE values varied among features, indicating different levels of overall causal influence across the features. For example, features like X10 started with a higher PEACE value at ‘*d’* = 0, suggesting a strong overall influence, which decreased more noticeably with increasing ‘*d’*.

**Figure 6:**
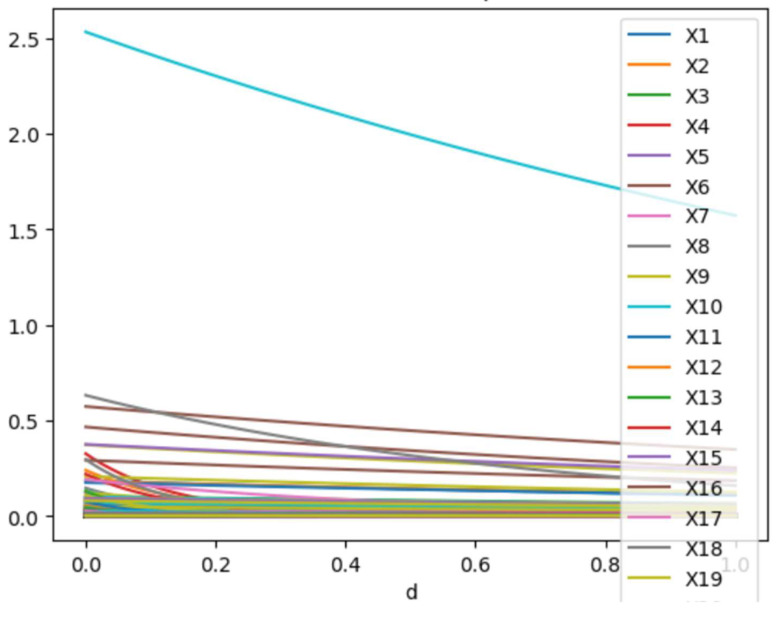
PEACE values for all columns for degrees d between 0 and 1.

Figure 6 provides a detailed demonstration of the PEACE values, highlighting that X10 had the highest total direct causal impact on the outcome. Please note that although X19 appears as the last element in the legend, this graph represents PEACE values for all elements of the dataset.

### 5.5. Mean Negative PEACE

**Figure 7:**
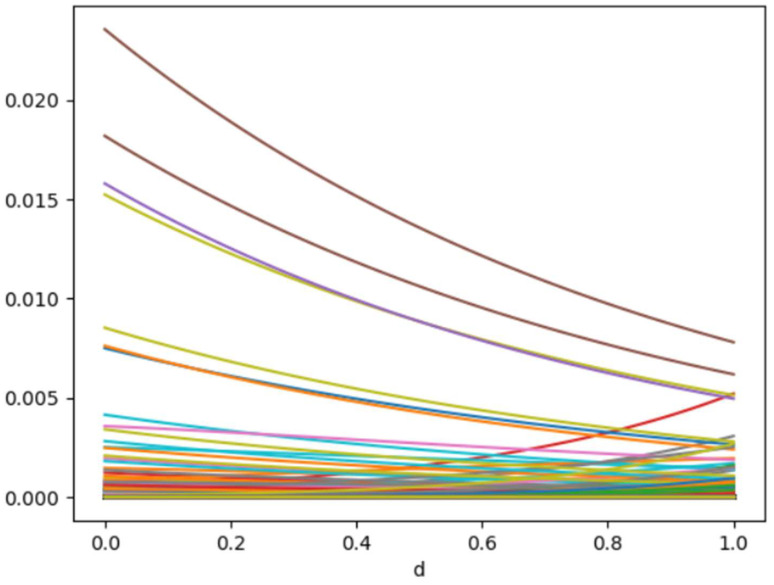
PEACE values for all columns in the dataset. The x-axis represents the degree *d* which ranged between 0 and 1. The y-axis represents the Mean negative PEACE value.

In Figure 7, a high mean negative PEACE value at *d = 0* indicates that the feature had a significant negative direct causal effect on the outcome when equal importance was given to the causal effects of both more probable and less probable regions. X216, which correspond to ‘log-sigma-5-0-mm-3D_glcm_Imc1’ in the dataset, had the highest initial negative PEACE value, suggesting that it had the strongest initial negative causal effect. 

The negative PEACE values for most of the features generally decreased as *d* increased. The increasing mean negative PEACE function indicates that the negative causal effects of the more probable regions were likely higher than those of the other regions.

### 5.6. Mean Positive vs. Mean Negative PEACE

**Figure 8:**
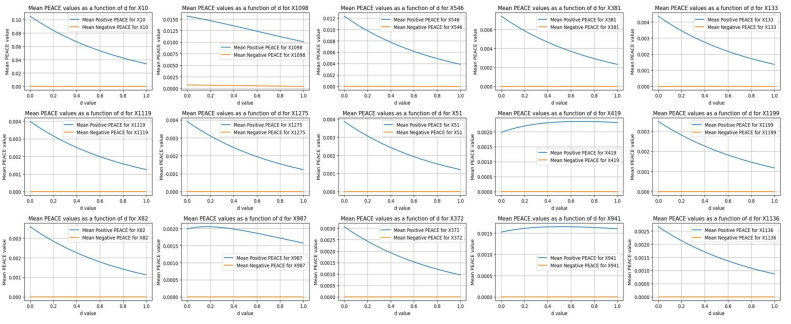
A detailed view of how the direct causal effects (both positive and negative) of various features change with the degree *d*.

Figure 8 compares the top mean positive PEACE values with their corresponding mean negative PEACE values for the features in the dataset. Each subplot illustrates how the positive (blue lines) and negative (orange lines) PEACE values of a specific feature changed as a function of the degree *d* from 0 to 1. 

The positive PEACE values showed higher initial values and a broader range, indicating stronger positive causal effects compared to negative effects. That is, while positive PEACE values decreased as *d* increased, the corresponding negative PEACE values remained close to zero, indicating relatively weak negative causal effects for these specific features. 

### 5.7. Mean Negative vs. Mean Positive PEACE

**Figure 9:**
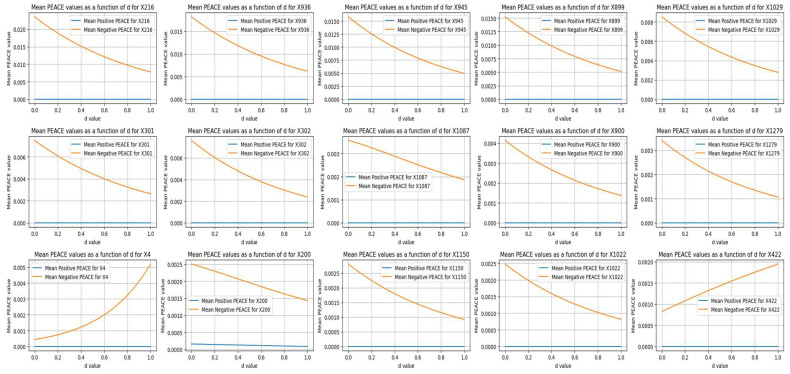
Comparison of the top negative PEACE values with their corresponding positive PEACE values for the leading features in the dataset.

Figure 9 compares the top mean negative PEACE values with their corresponding mean positive PEACE values for the leading features in the dataset. Each subplot illustrates how the positive (blue lines) and negative (orange lines) PEACE values of a specific feature change as a function of the degree *d* from 0 to 1.

The positive PEACE remained close to zero, indicating weak positive causal effects for these specific features. In contrast, the negative PEACE values started higher and decreased slightly as *d* increased, indicating more substantial negative causal effects. This comparison highlights the features that had significant negative influences on the outcome, with the corresponding positive influences being relatively minimal.

A comparison between Figure 8 and Figure 9 revealed that in this dataset, the positive PEACE values were generally higher in magnitude (e.g., a value of 0.10 for X10), while the negative PEACE values were relatively small (e.g., the highest value was 0.03 for X216). This indicates that the positive causal effects of the features on the outcome were much stronger compared to the negative causal effects. 

This comparison suggests that higher values of most radiomic features are causally associated with gliosarcoma rather than glioblastoma. In particular, a higher ‘original_shape_Sphericity’ of the tumor is causally associated with gliosarcoma and had the highest causal effect among the radiomic features in distinguishing between gliosarcoma and glioblastoma.

### 5.8. Comparison of SHAP, Marginal Effect, and ATE Results

Note that in our study, SHAP (SHapley Additive exPlanations) was used to explain the output of machine learning models by distributing the prediction among the input features. Although the results obtained by SHAP and PEACE both identified X10 as one of the most important elements in distinguishing gliosarcoma (GSM) from glioblastoma (GBM), SHAP is excellent for understanding and interpreting model predictions, while PEACE is more suitable for causal analysis, providing insights into the direct effects of features on outcomes. That is, SHAP can show correlations and, for different datasets, may show unrelated causes of the events (for more details please see [25]).

The PEACE/mean PEACE values provide a detailed gradient of causal effects at different levels of emphasis on data density, which was not directly visible in the marginal effect or ATE analyses. Furthermore, while ATE provided a constant measure of the treatment effect across the data, and the marginal effects showed how predictions change with feature values, PEACE offers a nuanced view that considers how the importance of different data regions (defined by their probability density) impacts causal estimation.

Also, the PEACE approach can highlight how certain features’ influences vary across different data concentrations, potentially offering insights into features that have significant effects in rare conditions (low ‘*d’* values) versus common conditions (high ‘*d*’ values). This contrasts with ATE, which averages out these effects, and marginal effects, which focus more on the functional relationship between feature changes and outcomes without explicitly considering data density.

In summary, the PEACE results provide a complementary perspective to ATE and marginal effects by explicitly incorporating the distributional characteristics of the data into the causal analysis. This approach can be particularly valuable in contexts were understanding the impact of common versus rare conditions is crucial, such as in healthcare or risk assessment.

Once more, we wish to underscore the methodology distinction. In prior research, including the work referenced in [20], the standard practice involves the initial application of dimension reduction techniques followed by using statistical and machine learning algorithms, such as LASSO, to perform causal inference. In contrast, our approach for this study involved the direct use of the entire dataset with PEACE, without any alterations or preprocessing steps.

## 6. Conclusions

Unlike previous studies on gliosarcoma detection using ultra-high dimensional confounders that relied on statistical models to reduce the size of the dataset before using machine learning algorithms for causal inference, our approach used the entire dataset.

In this study, we used Probabilistic Easy Variational Causal Effect (PEACE) [14] with the XGBoost [18], or eXtreme Gradient Boosting, algorithm.

Grounded in measure theory, PEACE offers a mathematical framework for both continuous and discrete variables. PEACE provides a spectrum of causal effect values based on the degree *d*, reflecting changes from natural to interventionally altered probability distributions. The model is stable under small changes in the partial derivatives of the causal function and the joint distribution of variables. Additionally, PEACE offers positive and negative causal effects, allowing for detailed analyses in both directions. It adjusts for the natural availability of values ranging from rare to frequent using a normalizer term and a degree *d*, ensuring the appropriate scaling and interpretation of causal effect.

In our study, we found that a higher ‘original_shape_Sphericity’ of the tumor was associated with gliosarcoma. This radiomic feature demonstrated the highest causal effect in differentiating between gliosarcoma and glioblastoma, highlighting its potential as a critical biomarker for accurate tumor classification.

Currently, the PEACE formula does not account for the temporal aspects of events. Our future work will focus on integrating temporality into the PEACE model to enhance its analytical capabilities. To address the intrinsic imprecision and uncertainty inherent in various real-world problems, our future work will involve integrating our causal framework with fuzzy logic. This integration aims to enhance the robustness and adaptability of our models, enabling them to handle ambiguity and partial truth values more effectively.

## Data Availability

The original data presented in the study are openly available at https://www.frontiersin.org/journals/oncology/articles/10.3389/fonc.2021.699789/full#supplementary-material (accessed on 27 June 2024).

## References

[1] Miller C.R., Perry A. (2007). Glioblastoma: Morphologic and molecular genetic diversity. Arch. Pathol. Lab. Med..

[2] Ohgaki H. (2009). Epidemiology of brain tumors. Cancer Epidemiol. Modif. Factors.

[3] Zaki M.M., Mashouf L.A., Woodward E., Langat P., Gupta S., Dunn I.F., Wen P.Y., Nahed B.V., Bi W.L. (2021). Genomic landscape of gliosarcoma: Distinguishing features and targetable alterations. Sci. Rep..

[4] Ammari S., Sallé de Chou R., Assi T., Touat M., Chouzenoux E., Quillent A., Limkin E., Dercle L., Hadchiti J., Elhaik M. (2021). Machine-learning-based radiomics MRI model for survival prediction of recurrent glioblastomas treated with bevacizumab. Diagnostics.

[5] Yang Y., Fan W., Gu T., Yu L., Chen H., Lv Y., Liu H., Wang G., Zhang D. (2021). Radiomic features of multi-ROI and multi-phase MRI for the prediction of microvascular invasion in solitary hepatocellular carcinoma. Front. Oncol..

[6] Yi X., Cao H., Tang H., Gong G., Hu Z., Liao W., Sun L., Chen B.T., Li X. (2019). Gliosarcoma: A clinical and radiological analysis of 48 cases. Eur. Radiol..

[7] Qian Z., Zhang L., Hu J., Chen S., Chen H., Shen H., Zheng F., Zang Y., Chen X. (2021). Machine learning-based analysis of magnetic resonance radiomics for the classification of gliosarcoma and glioblastoma. Front. Oncol..

[8] Jaman A., Wang G., Ertefaie A., Bally M., Lévesque R., Platt R., Schnitzer M. (2024). Penalized G-estimation for effect modifier selection in the structural nested mean models for repeated outcomes. arXiv.

[9] Gao Q., Zhang Y., Sun H., Wang T. (2022). Evaluation of propensity score methods for causal inference with high-dimensional covariates. Brief. Bioinform..

[10] Liu Y., Gao Q., Wei K., Huang C., Wang C., Yu Y., Qin G., Wang T. (2024). High-dimensional generalized median adaptive lasso with application to omics data. Brief. Bioinform..

[11] Martin P., Holloway L., Metcalfe P., Koh E.-S., Brighi C. (2022). Challenges in Glioblastoma Radiomics and the Path to Clinical Implementation. Cancers.

[12] Tang D., Kong D., Pan W., Wang L. (2023). Ultra-high dimensional variable selection for doubly robust causal inference. Biometrics.

[13] Yu D., Wang L., Kong D., Zhu H. (2022). Mapping the genetic-imaging-clinical pathway with applications to alzheimer’s disease. J. Am. Stat. Assoc..

[14] Faghihi U., Saki A. (2023). Probabilistic Variational Causal Effect as A new Theory for Causal Reasoning. arXiv.

[15] Faghihi U., Saki A. (2024). Probabilistic Easy Variational Causal Effect. arXiv.

[16] Camille J. (1881). Sur la serie de Fourier. Camptesrendushebdomadaires Séances L’academie Des Sci..

[17] Faghihi U., Kalantarpour C., Saki A. (2022). Causal Probabilistic Based Variational Autoencoders Capable of Handling Noisy Inputs Using Fuzzy Logic Rules. Proceedings of the Science and Information Conference.

[18] Chen T., Guestrin C. Xgboost: A scalable tree boosting system. Proceedings of the 22nd Acm Sigkdd International Conference on Knowledge Discovery and Data Mining.

[19] Pearl J., Mackenzie D. (2018). The Book of Why: The New Science of Cause and Effect.

[20] Faghihi U., Robert S., Poirier P., Barkaoui Y. From Association to Reasoning, an Alternative to Pearl’s Causal Reasoning. Proceedings of the AAAI-FLAIRS 2020.

[21] Janzing D., Minorics L., Blöbaum P. Feature relevance quantification in explainable AI: A causal problem. Proceedings of the International Conference on Artificial Intelligence and Statistics.

[22] Janzing D., Balduzzi D., Grosse-Wentrup M., Schölkopf B. (2013). Quantifying causal influences. Ann. Statist..

[23] Shapley L.S. (1953). A value for n-person games. Contrib. Theory Games.

[24] Chernozhukov V., Chetverikov D., Demirer M., Duflo E., Hansen C., Newey W., Robins J. (2018). Double/debiased machine learning for treatment and structural parameters. Econom. J..

[25] Lundberg S., Dillon E., LaRiviere J., Roth J., Syrgkanis V. (2021). Be careful when interpreting predictive models in search of causal insights. Towards Data Sci.

